# A hundred-year-old mystery—the reproductive mode and larval morphology of the enigmatic frog genus *Allophryne* (Amphibia; Anura; Allophrynidae)

**DOI:** 10.1007/s00114-024-01910-y

**Published:** 2024-04-10

**Authors:** Pedro Henrique dos Santos Dias, Jesse Delia, Carlos Taboada, Ronald Altig, Marco Rada

**Affiliations:** 1 Leibniz Institut Zur Analyse Des Biodiversitätswandels, Zoologisches Museum Hamburg, Zentrum Für Taxonomie Und Morphologie, Martin-Luther-King-Platz 3, 20146 Hamburg, Germany; 2 Department of Herpetology, American Museum of Natural History, New York, NY USA; 3 Department of Biological Sciences, Vanderbilt University, Nashville, TN USA; 4 Department of Biological Sciences, Mississippi State University, Mississippi State, MS USA; 5 Departemento del Quindío, Programa de Biología, Universidad del Quindío, Armenia, Colombia

**Keywords:** Centrolenidae, Reproductive biology, Evolution, Musculo-skeletal system, Systematics, Tadpoles

## Abstract

Frogs of the Allophrynidae are an enigmatic family from South America. To date, published information is lacking regarding this group’s reproductive biology and larval morphology. Here, we provide the first detailed description of the reproductive mode, developmental mode, and tadpole morphology for *Allophryne ruthveni*. We developed a captive breeding and rearing protocol for this species and then conducted a series of observations to describe aspects of its reproductive biology. In captivity, this species exhibits aquatic oviposition, where single eggs are laid ungrouped within a simple jelly capsule and are scattered free in the water column before sinking to develop on benthic substrates. We did not observe parental care nor any parental interactions with eggs post-fertilization. Tadpoles are characterized by an oval body, anteroventral oral disc, a labial tooth row formula of 2(2)/3, and a dextral vent tube. The buccopharyngeal cavity is marked by the presence of two pairs of infralabial papilla and four lingual papillae. Cranial morphology is characterized by the presence of the commissura quadratoorbital. This species possesses an additional slip of the m. rectus cervicis and of the m. levator arcuum branchialium III. We discuss our results in comparison with glassfrogs (Centrolenidae).

**Supplementary Information:**

The online version contains supplementary material available at 10.1007/s00114-024-01910-y.

## Introduction

The Allophrynidae is among the most enigmatic groups of frogs in the world. This family currently consists of just three species, two of which were described within the last 12 years (Gaige 1926; Castroviejo-Fisher et al. 2012; Caramaschi et al. 2013). Very little is known about these groups’ reproductive biology. In fact, of the 58 recognized families of frogs worldwide (sensu Frost 2024), the Allophrynidae is the only remaining family that lacks basic information about their reproductive mode, developmental mode, and larvae (Altig 2018). Numerous independent studies have recovered the Allophrynidae as the sister family to glassfrogs of the Centrolenidae (e.g., Frost et al. 2006; Guayasamin et al. 2008; Pyron and Wiens 2011; Twomey et al. 2014; Streicher et al. 2018). Recently, there has been increased interest in understanding the evolution of reproductive, larval, and coloration traits within this group (Haas 2003; Hoffmann 2010; Delia et al. 2017; Escalona Sulbarán et al. 2019; Rada et al. 2019; Dias et al. 2020; Taboada et al. 2020, 2022; Montilla et al. 2023). Documenting reproductive traits for allophrynids will contribute to our understanding of this family’s biology, as well as facilitate comparative research with Centrolenids and other frogs.

To date, information on reproduction in allophrynid frogs is largely limited to anecdotal observations of explosive breeding events. All three species of *Allophryne* appear to be explosive breeders, with reports of chorusing males encountered calling from arboreal vegetation at night around forest ponds, flooded forest, and streams (Caldwell 1996; Duellman 1997; Caramaschi et al. 2013; Fonseca et al. 2022). These breeding events occurred after heavy rainstorms and, in at least *A. ruthveni*, breeding appears to be limited to a few larger storms per year (Gottsberger and Gruber 2004; Caramaschi et al. 2013; Fonseca et al. 2022; MA Rada pers. obs.). Based on their association with aquatic environments during explosive breeding events, it is thought that allophrynids exhibit indirect development and lay eggs in either aquatic or terrestrial/arboreal sites. However, published reports are convoluted. For example, Duellman (1997) inferred aquatic oviposition in *A. ruthveni* based on a collected pair laying eggs in a plastic bag, whereas Lescure and Marty (2000) report that this species lays eggs on arboreal vegetation overhanging water (similar to centrolenids).

Information on allophrynid tadpoles is also limited and anecdotal. Lescure and Marty (2000) provided a single-sentence tadpole description of *A. ruthveni* as “Tadpole flattened dorsoventrally, grey-brown with black spots, tail pointed,” without any reference to collected materials or supporting information (i.e., how species identity was confirmed). Gottsberger and Gruber (2004) monitored the tadpole phenology of a community of frogs from French Guiana, including *A. ruthveni*, but they did not report this species’ larval description, how/whether tadpole identity was confirmed, nor reference any collected material that is publicly available.

Here, we provide the first detailed description of the reproductive mode, developmental mode, and tadpole morphology for a member of the Allophrynidae, *Allophryne ruthveni*—a species discovered over 97 years ago from Guyana (Gaige 1926). We describe the reproductive mode of this species by conducting observations in captivity. We also described the tadpole of *A. ruthveni*, including its cranium, buccal cavity, and musculature anatomy. Finally, we discuss our results in comparison with available information on centrolenids and other frogs.

## Material and methods

### Reproductive biology

#### Captive frogs and breeding

We purchased a group of five adult wild-caught *Allophryne ruthveni* from the pet trade in the USA (4 males, 1 female) imported from Suriname. The exact collecting locality is not known. All captive maintenance, breeding, and tadpole rearing and collecting were conducted by J. Delia and C. Taboada. Research and colony care procedures were approved by the Institutional Animal Care and Use Committee of the American Museum of Natural History (#AMNHCIACUC-20220120) and Duke University (#A210-18–09 [78458] and A174-21–08).

We bred both wild-caught and captive-bred adults in captivity. We maintained frogs in bioactive vivaria, set to their native tropical conditions (temp 22–25 °C, RH ≥ 70%, 12 h light/dark cycles, frequent daily misting). We used a soil mix on top of a raised bottom and planted the vivarium with common *Epipremnum* and *Philodendron* trailings, and *Neoregelia* bromeliads. We added a layer of dry live oak leaves (*Quercus virginiana*) on top of the soil mix, along with some cork bark flats for cover, and seeded the tank with springtails (Collembola) to help break down frog waste. We fed frogs a mixed diet of small crickets and flightless fruit flies (*Drosophila hydei*) every 3–5 days.

Very little is known about this species’ reproduction, outside of being an explosive breeder that calls from vegetation around flooded forest, ponds, and swaps after heavy rainstorms (Caldwell 1996; Duellman 1997). Therefore, we constructed a rain chamber to simulate a heavy rainstorm over a flooded forest pool with a range of call- and oviposition-site options. The chamber was constructed out of a clear plastic tote with a gasket lid top (~ 38^H^ × 63^L^ × 45^W^ cm). The lid was lined with a flexible plastic hose (1.9 cm OD), organized in rows, drilled with small holes, and connected to a small submersible water pump (80 GPH) positioned on the floor of the chamber. Once flooded, water would pump from the bottom of the flooded tank up to the lid and drip back down through the hosing. To create options for call and oviposition sites, we vegetated the chamber with *Epipremnum* spp. and *Philodendron* spp. trailings and allowed the plants to develop root mats in the water bottom. We also added emergent stones and sticks, and cork bark, which along with root mats provided a range of oviposition sites above the water, at the surface, and under the water. We flooded the tank with ~ 12 cm of aged tap water and installed an aquarium heater set to 22–24 °C.

To breed the wild-caught group, we first increased the frequency of misting within their home vivarium (roughly doubling the frequency) for 1 week. We then cycled the group in the rain chamber for 3 days, removed and fed them in their home tank for 3 days, and then cycled them again for 3 more days. Males called frequently while in the rain chamber at night and often during the day. The group was returned to their home tank, and after a few weeks, developing eggs became visible through the female’s abdomen. We then cycled the group for 3 days, followed by 3 days of feeding in their home tank, and again cycled them in the rain chamber. After two rounds of cycling, the female paired up and laid eggs. Many of the resulting offspring were reared to adults and then bred. Captive-bred adults were easier to breed than the founding group, often laying during the first or second cycle in the rain chamber.

#### Rearing tadpoles and juvenile frogs

We allowed eggs to develop until hatching in the rain chamber and moved hatchlings to 10-gallon aquaria. The aquaria were set with a mix of aquarium stone and several dry almond leaves to each tank to provide leaf-litter-pack microhabitats on top of the stone layer (typical of tropical forest ponds). Each tank was equipped with a small sponge filter attached to a bubbler (set to low), an aquarium heater (set to 22–24 °C), and a bouquet of *Epipremnum* spp. clippings which rooted into the tank. We used aged (48 h) tap water to set tanks and for subsequent water changes. We initially fed tadpoles a range of food items, including boiled greens, boiled cod, fish flakes, turtle and cichlid food pellets, and fish meal gels. After finding that tadpoles eat all food items, we fed them a rotation of boiled greens and a mix of meal gels to target an omnivorous diet*.* Once tadpoles reached Gosner (1960) stages 41–42, we moved them to plastic deli cups consisting of flooded *Sphagnum* sp. moss, such that they had emergent substrate to haul out (*A. ruthveni* will drown if not provided with easy haul-out options—unlike glassfrogs, which haul out vertically on aquarium walls). After their tail was mostly reabsorbed (~ stage 45), we moved them to small vivarium set like adult tanks and fed springtails and fruit flies (*Drosophila melanogaster*).

#### Observations of reproduction, parental behavior, and offspring development

We conducted observations in captivity during six breeding events. Two of these events were for the same wild-caught female, and the additional four events were for independent pairs of captive-bred adults. We describe aspects of their reproductive and developmental mode (sensu Salthe and Duellman 1973; Wells 2007; Altig and McDiarmid 2007; Crump 2015). We cycled independent groups (1 female with 2–3 males) in the rain chamber and repeatedly checked them and/or video-recorded their behaviors. After oviposition, parents were left in the chamber until all eggs hatched or died. We conducted repeated observations of parents and their eggs to determine whether parents interact with eggs, the duration of embryonic development, and the onset of hatching. To do so, we repeatedly checked parents and their eggs at 30–60 min intervals over 4–6 h during the day and over 4 h at night throughout embryonic development (until all eggs hatched or died). The water temperature of the rain chamber during breeding and embryonic development was maintained between 22 and 24 °C.

## Tadpole morphology

We describe tadpole morphology using 15 individuals in developmental stages 26–30 (sensu Gosner 1960) housed in the herpetological collection of the Leibniz Institut zur Analyse des Biodiversitätswandels, Zoological Museum of Hamburg (ZMH 19224–19226). Additional specimens not included in our analyses are housed in the herpetological collection of the American Museum of Natural History. We also examined tadpoles of representatives of different species of centrolenids, allophrynids’ sister group, and of other closely related lineages (see Appendix 1 for the complete list of examined material) to understand character variation. Terminology for external morphology characters is that of Altig and McDiarmid (1999) and Altig (2007).

### Buccopharyngeal cavity

We studied the buccopharyngeal cavity of two individuals at stage 30 (ZMH 19224). Tadpoles were manually dissected according to Wassersug (1976). After inspection under a stereoscopic microscope, one of them was prepared according to the protocol of Dias and Anganoy-Criollo (2024) for scanning electron microscopy (SEM). Terminology follows Wassersug (1976, 1980).

### Musculo-skeletal system

We submitted three tadpoles at stages 28–30 (ZMH 19226) to the clearing and double-staining protocol of Dingerkus and Uhler (1977). The protocol was interrupted after the cartilages were stained with alcian blue solution, the individuals were manually dissected, and the muscles were stained with Lugol solution to aid visualization. After inspection of the origin and insertion of all larval muscles, the process of clearing was completed for the study of the chondrocranium. Terminology for muscles and skeleton is that of Vera Candioti et al. (2024).

### Character evolution

We reconstructed the evolutionary history of some characters (see Appendix 1) to understand how the larvae of Allophrynidae diversified. We examined representatives of the clade Allophrynidae + Centrolenidae and from closely related lineages (e.g., Leptodactylidae, Bufonidae). Allophrynidae + Centrolenidae have been frequently recovered as the sister clade to Leptodactylidae in phylogenetic hypotheses with dense taxon sampling (Frost et al. 2006; Pyron and Wiens 2011; Jetz and Pyron 2018; Portik et al. 2023). Our taxon sampling was based on the phylogenetic hypothesis of Jetz and Pyron (2018). We constructed a character matrix using Mesquite V. 3.51 (Maddison and Maddison 2018) and used parsimony optimization (Fitch 1971) performed with T.N.T.v. 1.5 (Goloboff and Catalano 2016). Our goal is to identify major evolutionary patterns, but future studies with a more complete sampling within the clade Allophrynidae + Centrolenidae will be necessary to test our propositions. Given the lack of information for the larvae of the other two species of *Allophryne*, apomorphic transformations are treated as putative synapomorphies.

## Results

### Reproductive mode (Fig. 1A–H)

**Fig. 1:**
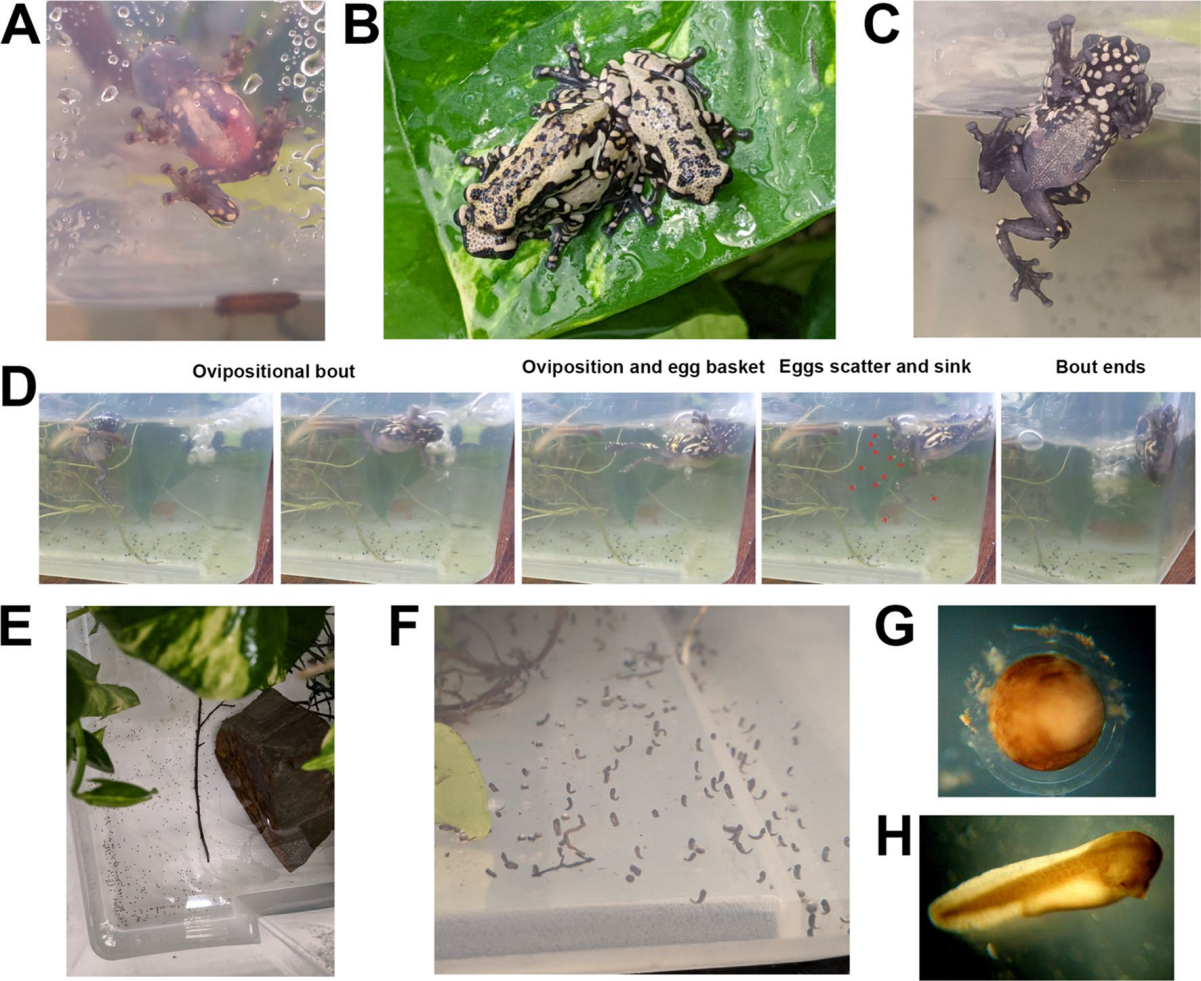
Reproduction in *Allophryne ruthveni*. **A** A male calling from the side of the rain chamber just above the water line. **B** and **C** Pairs in amplexus. Amplexus began at arboreal sites, after which the pair eventually moved into the water to perform an ovipositional bout. **D** A sequence of a single ovipositional bout (see Supp. Video 1). The pair (female) swam from the side of the tank into the water, during which the female oviposited several eggs that were captured by the male using his feet forming an “egg basket” (to presumably fertilize the eggs). The individual eggs (red arrows) then scattered in the parents’ swim strokes and sank to the bottom of the tank, after which, the ovipositional bout ended. In all six breeding events, parents performed numerous ovipositional bouts throughout the rain chamber over several hours until 100 s of eggs were scattered across the bottom of the tank and on submerged substrates. **E** A portion of a clutch laid by a single pair, with individual eggs scattered free across the bottom of the rain chamber. **F** embryos began to hatch ~ 30 h post oviposition. **G** *A. ruthveni* lays individual eggs in a simple capsule (note: two obvious jelly layers visible using brightfield microscopy—additional approaches are required to better examine and count jelly layers). **H** Hatching occurs at Gosner (1960) stages 18–19, at the onset of a muscular response and a heartbeat). All images were taken of animals in captivity

In captivity, we observed that *Allophryne ruthveni* exhibits an aquatic reproductive mode, involving aquatic egg-laying, external fertilization during axillary amplexus, aquatic embryonic development, an aquatic free-living larval stage, and a lack of post-fertilization parental care. During all six breeding events, females laid large clutches of several hundred individual eggs that were scattered free throughout the tank bottom (Fig. 1, Supp. Video 1). We were unable to count the total clutch size without disturbing embryos, as individual eggs were scattered over all surfaces under water including stones, sticks, and root mats. However, all females laid 100 s of individual eggs that were oviposited in small groups during numerous ovipositional bouts while in amplexus with the same male.

Amplexus and ovipositional bouts began at night and occurred over several hours, with three pairs continuing well into daylight (until ~ 16:00 h). During this time, pairs remained in axillary amplexus, and the pair (female) moved around arboreal vegetation and the chamber walls in between ovipositional bouts (Fig. 1A–C). During each ovipositional bout (Fig. 1D, Supp. Video 1), the pair approached the water’s edge and partially entered the water, where they remained for a brief period. The pair (female) then swam out into the open water and immediately oviposited a small group of eggs just under the water surface, during which she exhibited trunk-muscle contractions with her back arched and limbs extended. As the eggs exited the females’ cloaca, the male briefly caught them by coordinating an “egg basket” with his hind feet (presumably to fertilize the eggs) and immediately released them. The eggs immediately scattered in the wake of the parents’ swim strokes and sank to the bottom (i.e., parents did not attach them to substrates) (see Supp. Video 1). Following each ovipositional bout, the pair (female) swam to the emergent substrate, where they either remained partially submerged or moved back up into the vegetation/chamber wall and to a different section of the enclosure. After a period (minutes to 10 s of minutes), they performed another bout of aquatic egg laying. This general sequence was repeated over several hours, such that the pair deposited groups of eggs throughout the water area, and eggs were scattered over the entire bottom of the rain chamber (including on the plastic bottom, on submerged rocks, sticks, and root mats), without any obvious pattern for egg deposition sites (Fig. 1E). Fertilization success and embryo survival until hatching appeared to be high for six independent clutches, as we observed very few undeveloped and/or dead eggs (Fig. 1F).

#### Parental care observations

We did not observe any evidence that *A. ruthveni* exhibits parental interactions with embryos following oviposition. During repeated checks for six pairs, the parents did not interact with their eggs during development, nor did they associate with the same habitat as embryos during development. All parents remained in arboreal sites above the water while eggs developed underwater on the bottom of the tank.

#### Eggs

*Allophryne ruthveni* oviposits single, ungrouped eggs, within a simple jelly capsule. Under a dissecting microscope, we noted that the egg capsule appears to consist of two clearly defined jelly layers (Fig. 1G)—however, additional approaches will be required to better document and count the structural layers of allophrynid eggs. The ova are dark-pigmented on the animal pole and unpigmented on a vegetal pole. Egg-capsule morphology is simple and characteristic of aquatic types—the capsules do not remain turgid in air, and they deform unless submerged under water (Altig and McDiarmid 2007). We observed that eggs were deposited free and individually scattered just below the water surface where they immediately sank to the bottom and lightly adhered to benthic substrates.

#### Embryonic and larval development

In all six clutches, embryonic development occurred over 30–36 h (with water temps maintained at ~ 22–24 °C) (Fig. 1F–H). Hatching occurred at Gosner (1960) stages 18–19 (muscular response–heartbeat/gill buds, Fig. 1H). We could not confirm the exact time of oviposition, as eggs are scattered in bouts over several hours. Thus, we estimated the duration of embryonic development starting from 00:00 on the night/morning of oviposition.

Larval development occurred over 3–6 weeks (water temps maintained at 22–24 °C), with most individuals completing metamorphosis and hauling out of the water during weeks 3–4. After the onset of feeding competence, tadpoles quickly became voracious and swarmed food items both day and night. We found it necessary to continually increase the quantity of food with development, as well as the frequency of 50% water changes (from once a week to roughly every 2 days), to keep with optimal growth and reduce the level of waste. When not feeding, tadpoles rested on the bottom of the tank on top of the rock substrate layer, under almond leaves, or on the tank walls during the day and were active within the water column by night.

## Tadpole morphology

### External morphology (Figs. 2, 3, and 4)

**Fig. 2:**
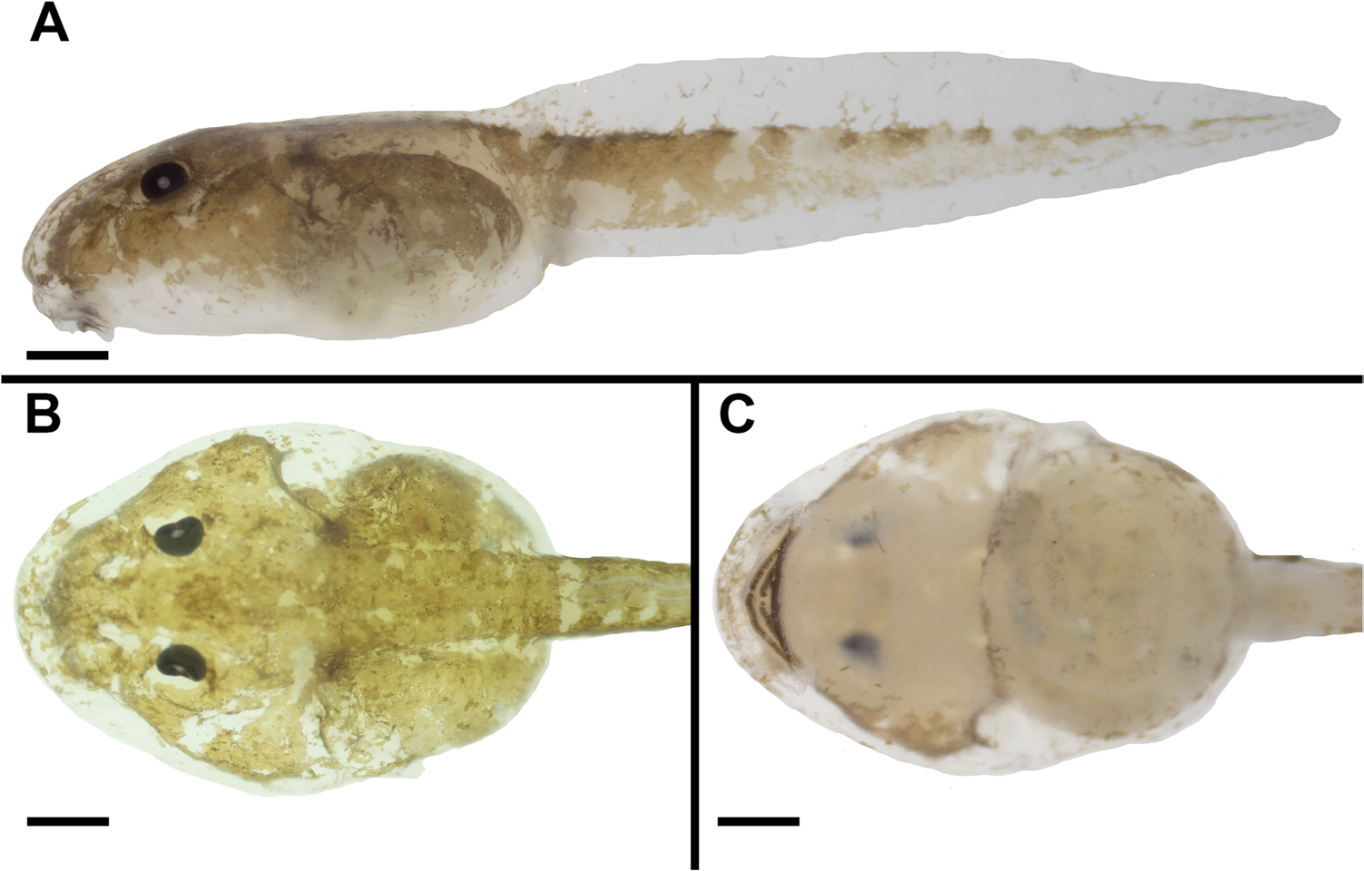
The tadpole of *Allophryne ruthveni* (ZMH 19225) at stage 29 in lateral (**A**), dorsal (**B**), and (**C**) ventral views. Scale bar = 0.5 mm

**Fig. 3:**
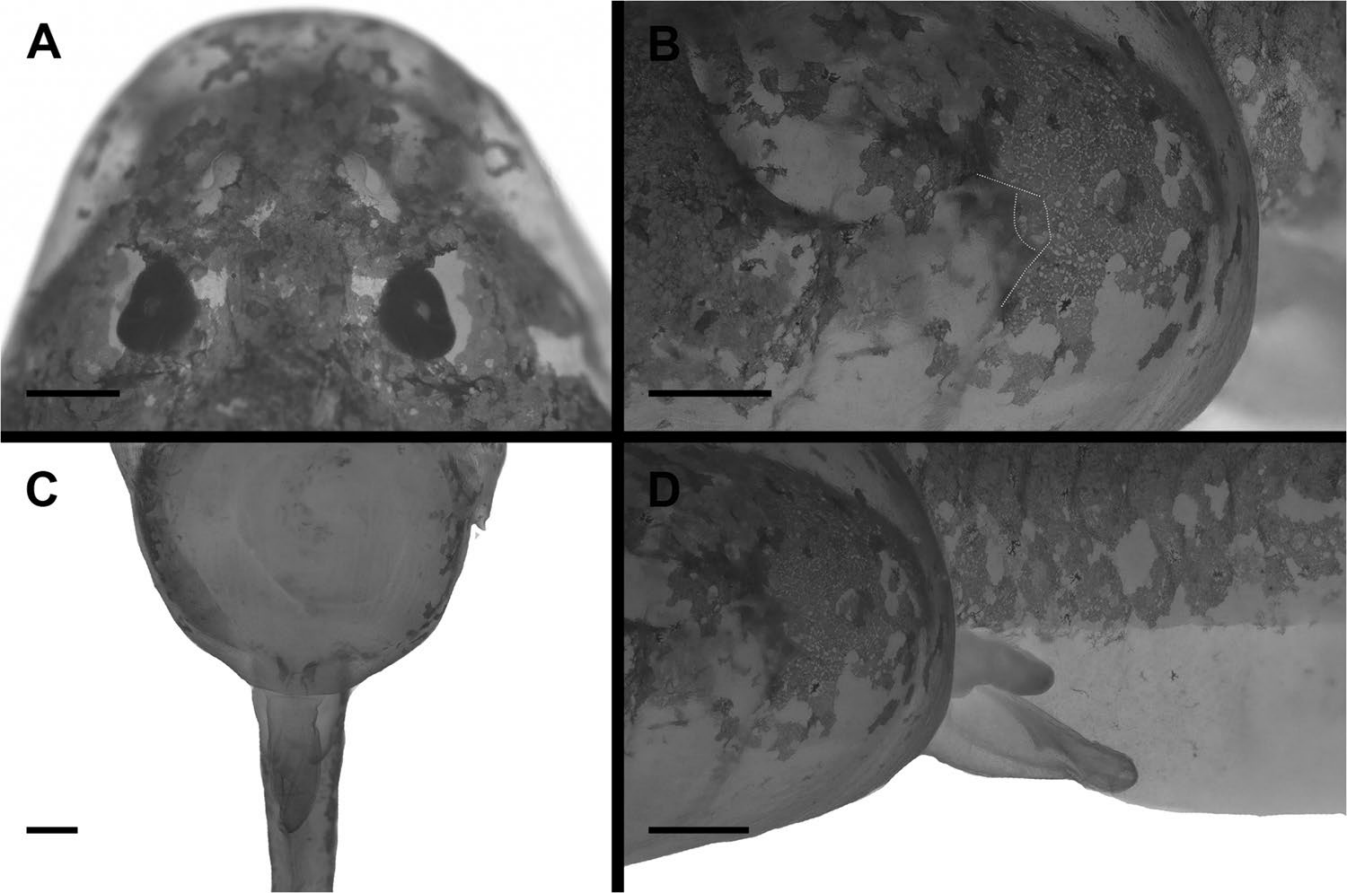
The tadpole of *Allophryne ruthveni* (ZMH 19225) at stage 29; details of the snout and nostrils (**A**), sinistral spiracle (highlighted, **B**), and sinistral vent tube in ventral (**C**) and **D** lateral views. Scale bar = 0.5 mm

**Fig. 4:**
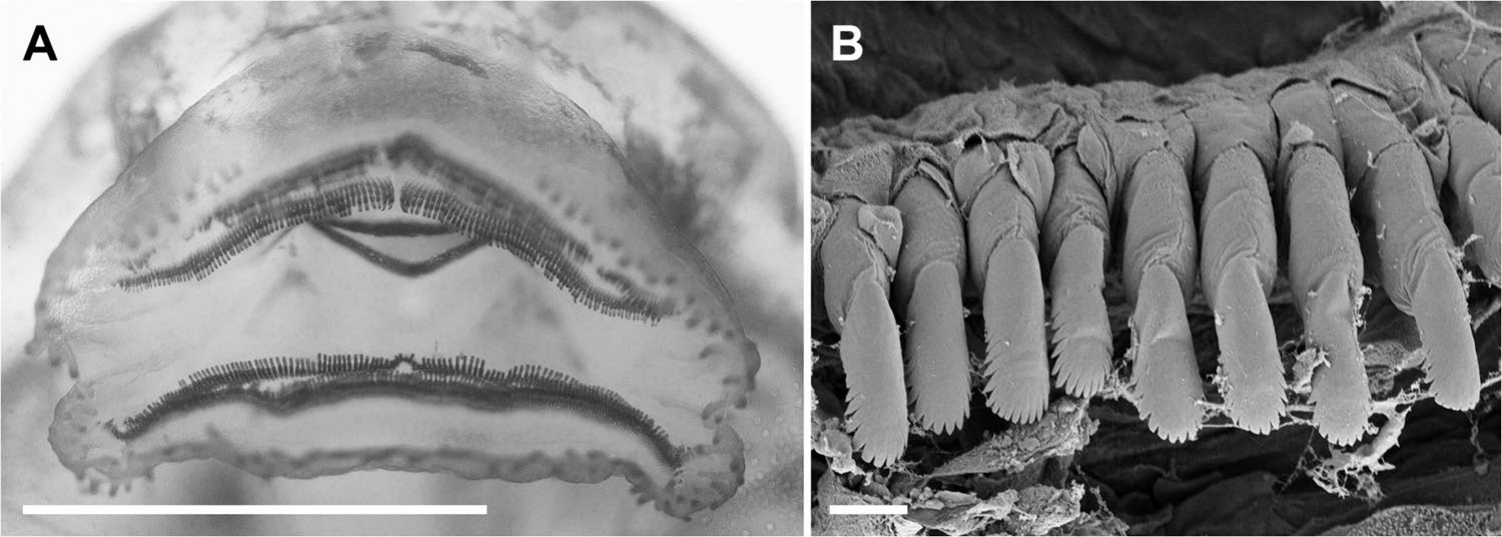
The oral apparatus of the tadpole of *Allophryne ruthveni*; oral disc (**A**) at stage 29 (ZMH 19225) and details of labial teeth (**B**) at stage 30 (ZMH 19224). Scale bars = 1.0 mm (**A**) and 10 µm (**B**)

Body elliptical in dorsal view, snout rounded, slightly rhomboid (Fig. 2). Eyes dorsal, dorsolaterad. In lateral view, body cylindrical, snout rounded. Nares enlarged (Fig. 3A), elliptical, with marginal rim. Spiracle sinistral, tubular, lateral, distally free from the body (Fig. 3B). Vent tube sinistral, tubular (Fig. 3C, D). Tail long, with rounded tip (Fig. 2A); dorsal fin originates at the body/tail junction. Oral disc (Fig. 4A) ventral, laterally emarginate, bordered by a single row of conical, marginal papillae; medial diastema in upper lip present. Submarginal papillae absent. Labial tooth row formula (LTRF) 2(2)/3; A1 and A2 lengths subequal; A2 interrupted with small gap; P1 and P2 lengths subequal, longer than P3; oral disc translucent, free of pigmentation. Each tooth (Fig. 4B) has a body and a head well-defined; cusps present. Upper jaw sheath arched, strongly keratinized in the border, finely serrated; lower jaw sheath V-shaped, strongly keratinized in the border, finely serrated. Lateral line stiches discreet; stiches in the supraorbital, infraorbital, dorsal trunk, middle, and trunk lines present.

### Buccopharyngeal cavity (Figs. 5 and 6)

**Fig. 5:**
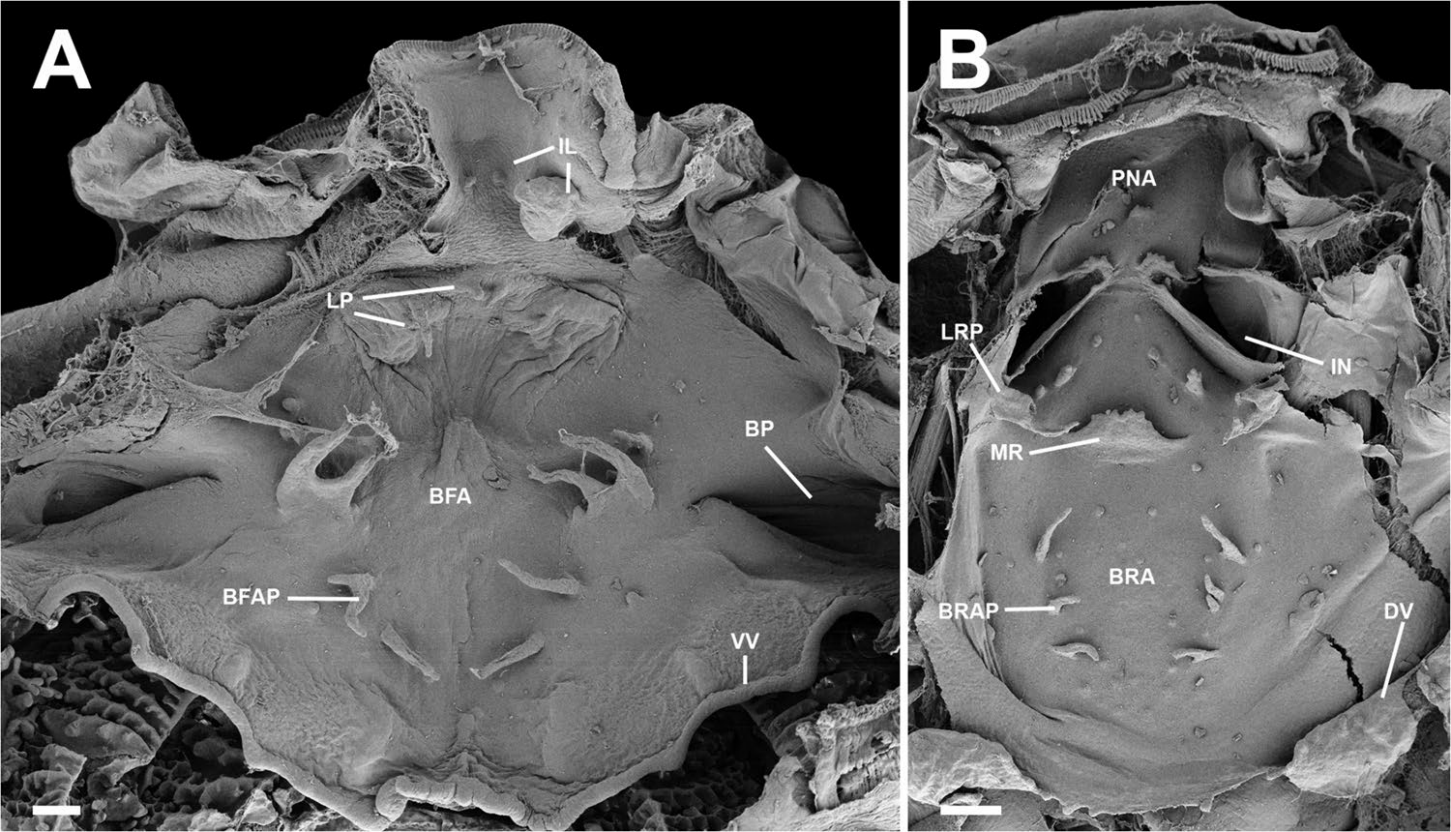
Buccopharyngeal cavity of the tadpole of *Allophryne ruthveni* (ZMH 19224) at stage 30; buccal floor (**A**) and buccal roof (**B**). BFA, buccal floor arena; BFAP, buccal floor arena papillae; BP, buccal pocket; BRA, buccal roof arena; BRAP, buccal roof arena papillae; DV, dorsal velum; IL, infralabial papillae; IN, internal nare; LP, lingual papillae; LRP, lateral ridge papillae; MR, median ridge; PNA, prenarial arena; VV, ventral velum. Scale bar = 100 µm

**Fig. 6:**
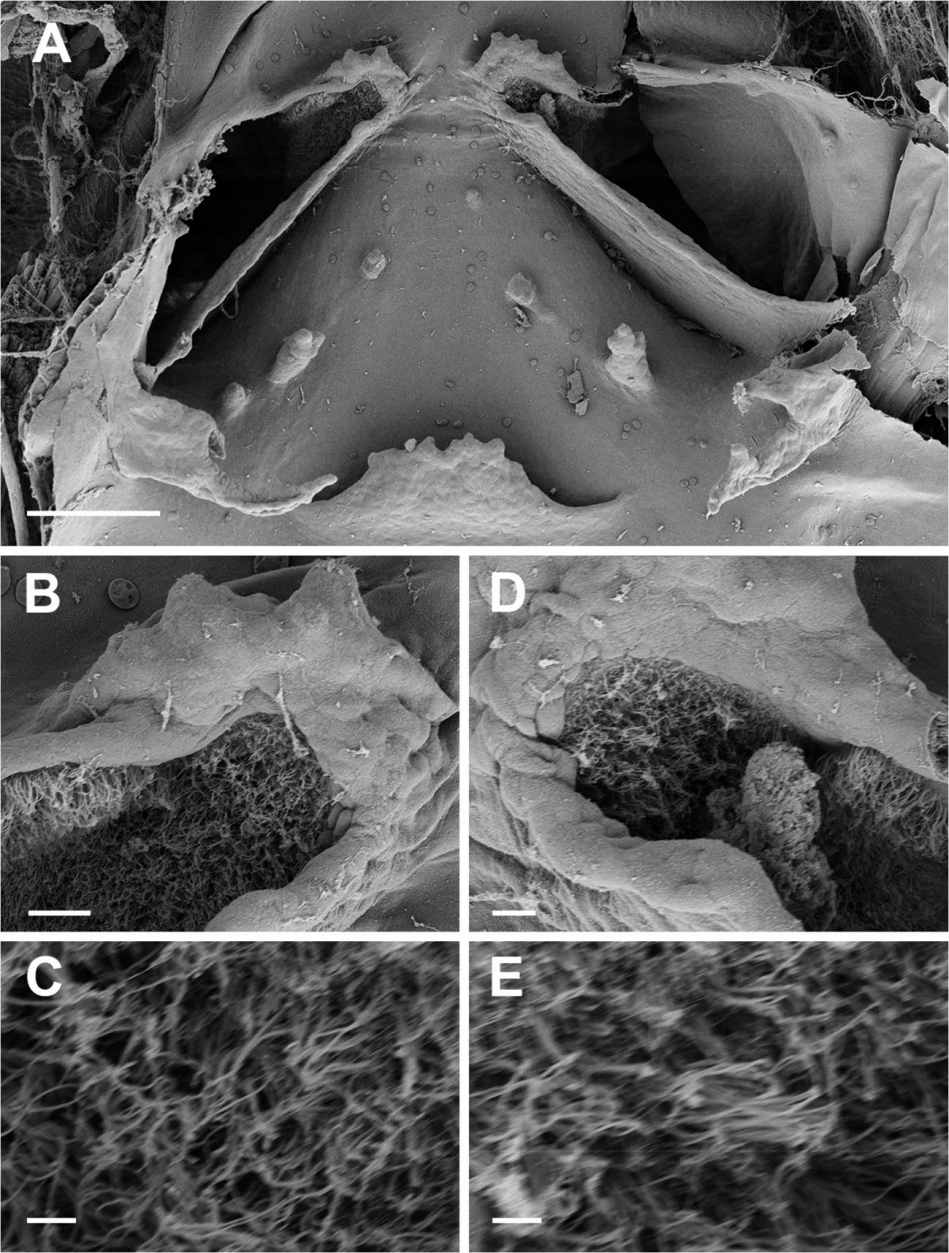
Buccopharyngeal cavity of the tadpole of *Allophryne ruthveni* (ZMH 19224) at stage 30; details of the internal nares (**A**); ciliated fields inside the right (**B** and **C**) and left (**D** and **E**) nares. Scale bars = 100 µm (**A**), 10 µm (**B** and **D**), and 1 µm (**C** and **E**)

Buccal floor triangular (i.e., narrow anteriorly, wide posteriorly; Fig. 5A). Two pairs of infralabial papilla (Fig. 5A); medial pair short, conical; lateral pair short, large, and globose. Tongue anlage cylindrical, bearing four conical, lingual papillae (Fig. 5A); central pair shorter than lateral pair. Buccal floor arena U-shaped, laterally delimited by six papillae, with few pustulations; papillae at the level of the buccal pocket bifurcated. Single conical, pre-pocket papilla present. Buccal pockets obliquely oriented, deep, perforated. Ventral velum with evident spicular support, arch-shaped, with irregular margin, with small, marginal projections; discreet secretory pits scattered along the margin. Medial notch present (not evident in Fig. 5) and well-marked; glottis exposed. Branchial basket triangular shallow, bearing three evident filter cavities.

Buccal roof triangular (Fig. 5B) longer than wide. Prenarial arena long, rhomboid, with a small protuberance. Internal nares elliptical (Fig. 6A), arranged obliquely to the anteroposterior axis; prenarial papillae absent; narial valve poorly developed or absent. Anteromedial portion of the internal nares covered by a ciliated epithelium (Fig. 6B–E). Postnarial arena rectangular, bearing two small, conical postnarial papillae; few, scattered, round pustulations present. Median ridge trapezoid, low, irregular margin. Lateral ridge papillae long, bifurcated. Buccal roof arena U-shaped, delimited by 3–4 buccal roof arena papilla each side; few, rounded pustulation present. Glandular zone evident, with scattered secretory pits. Dorsal velum arched, with smooth margin, interrupted medially, lacking papillae.

### Visceral components

Coiled gut with switchback points sinistral. Intestines long, unpigmented, with regular diameter (i.e., no dilatation of the anterior or posterior portions). Lungs reduced, present as poorly developed buds, not inflated (absent, sensu Haas 2003). Sinus branchialis (sensu Hoffmann 2010) absent.

### Larval muscles (Fig. 7)

**Fig. 7:**
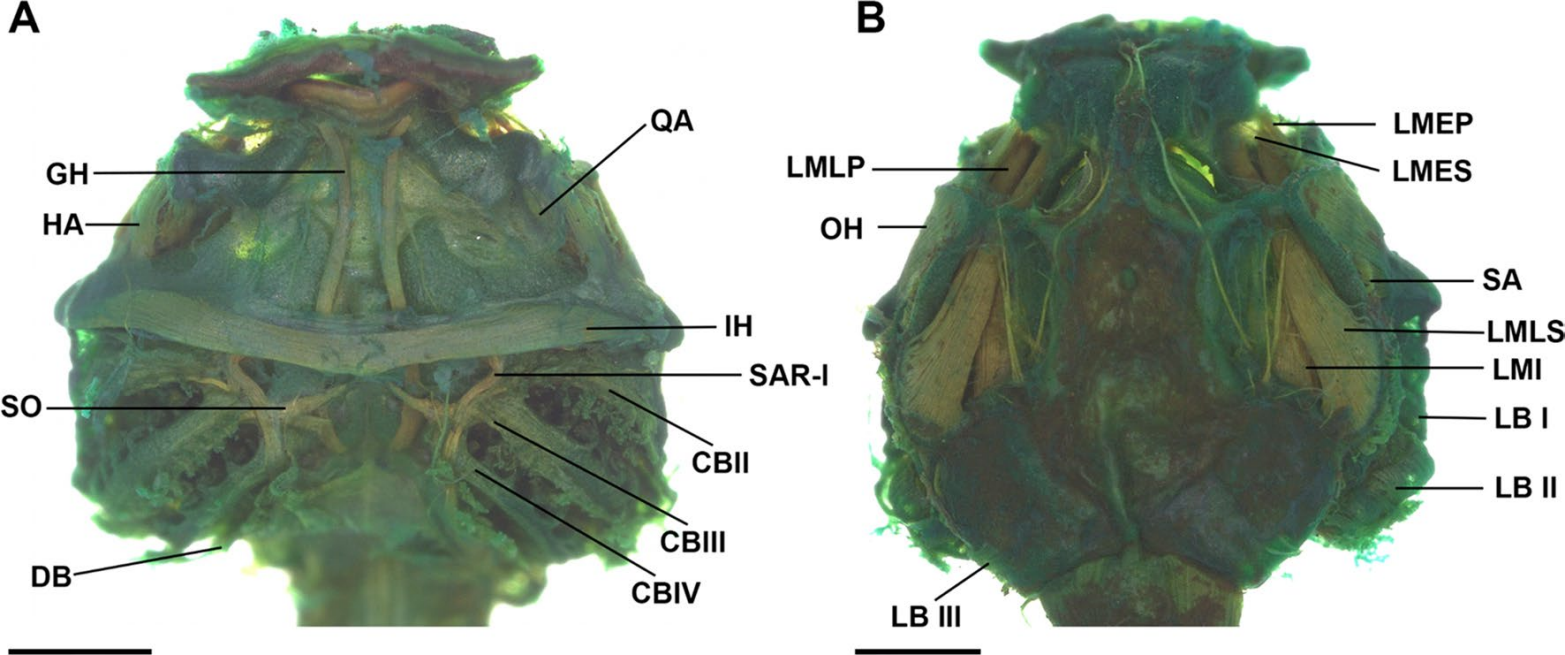
Larval muscles of the tadpole of *Allophryne ruthveni* (ZMH 19226) at stage 30 in ventral (**A**) and dorsal (**B**) views. CB (I–IV), constrictor branchialis; DB, diaphragmatobranchialis; GH, geniohyoideus; HA, hyoangularis; IH, interhyoideus; LB (I–III), levator arcuum branchialium; LMEP, levator mandibulae externus profundus; LMES, levator mandibulae externus superficialis; LMI, levator mandibulae internus; LMLP, levator mandibulae longus profundus; LMLS, levator mandibulae longus superficialis; OH, orbitohyoideus; QA, quadratoangularis; SA, suspensorioangularis; SAR I, subarcualis rectus I; SO, subarcualis obliquus. Scale bar = 0.5 mm

We detected 32 muscles in the larvae of *Allophryne ruthveni* (Table 1). Besides the muscles listed in Table 1, we also observed the presence of the superficial branchial muscle interhyoideus posterior; it is poorly developed and represented by some loose, spaced fibers. The most remarkable feature, however, is the presence of an additional slip of the levator arcuum branchialium III, originating dorsolateral in the otic capsule and inserting on the constrictor branchialis III. A secondary slip of the muscle rectus cervicis inserts on the ceratobranchial IV.

**Table 1:** Cranial, hyoid, and hyobranchial musculature of the tadpoles of *Allophryne ruthveni*

Muscle	Origin	Insertion	Comments
Mandibular group, n. trigeminus (c.n. V) innerved			
Levator mandibulae longus superficialis	External posterior margin arcus subocularis	Dorsomedial cartialgo Meckeli	Via long tendon
Levator mandibulae longus profundus	External margin (curvature) arcus subocularis	External margin of the suprarostral ala	Via a long tendon
Levator mandibulae externus superficialis	Inner processus muscularis (superior)	Adrostral tissue	c.n. runs above
Levator mandibulae externus profundus	Inner processus muscularis (medial)	Suprarostral alae	Share a tendon with LMLP
Levator mandibulae articularis	Inner processus muscularis (inferior)	Dorsal cartialgo Meckeli	
Levator mandibulae lateralis	Pars articularis quadrati	Adrostral tissue	
Submentalis (intermandibularis anterior)	-	-	
Intermandibularis	Median aponeurosis	Ventromedial cartialgo Meckeli	
Mandibulolabialis	Ventromedial Meckel`s cartilage	Lower lip	
Mandibulolabialis superior	-	-	
Levator mandibulae internus	Ventral processus ascendens and few fibers on the lateral curvature	Distal cartialgo Meckeli	
Hyoid group, n. facialis (c.n. VII)			
Hyoangularis	Dorsal ceratohyal	Retroarticular process of cartialgo Meckeli	
Quadratoangularis	Ventral palatoquadrate	Retroarticular process of cartialgo Meckeli	
Suspensorioangularis	Ventral palatoquadrate and posterior processus muscularis	Retroarticular process of cartialgo Meckeli	
Orbitohyoideus	Processus muscularis	Lateral edge of ceratohyal	
Suspensoriohyoideus	Posterior descending margin of processus muscularis	Lateral process of ceratohyal	
Interhyoideus	Median aponeurosis	Ventral ceratohyal	
Branchial group, n. Glossopharyngeus (c.n. IX) and vagus (c.n. X)			
Levator arcuum branchialium I	Arcus subocularis	Ceratobranchial I	
Levator arcuum branchialium II	Lateral capsula auditiva	Ceratobranchial II	
Levator arcuum branchialium III	Lateral capsula auditiva and dorsolateral capsula auditiva	Ceratobranchial III	Two slips
Levator arcuum branchialium IV	Capsula auditiva	Ceratobranchial IV	Separated from the TP
Tympanopharyngeus	Capsula auditiva	Ceratobranchial IV and pericardium	Separated from the LAB IV
Dilator laryngis	Capsula auditiva	Arytenoid cartilage	
Constrictor branchialis I	-	-	
Constrictor branchialis II	Processus branchialis II	Commissura terminalis I	
Constrictor branchialis III	Processus branchialis II	Commissura terminalis II	
Constrictor branchialis IV	Ceratobranchial III	Commissura terminalis II	
Subarcualis rectus I	Posterior lateral base of ceratohyal	Processus branchialis II and III and ceratobranchial I	
Subarcualis rectus II-IV	Ceratobranchial IV	Ceratobranchial II	
Subarcualis obliquus II	Processus urobranchialis	Ceratobranchial II and III	Two slips
Diaphragmatobranchialis	Peritoneum (diaphragm)	Distal Ceratobranchial III	
Spinal group, spinal nerve innervation			
Geniohyoideus	Hypobranchial plate	Cartialgo infrarostralis	At the level of CB III
Rectus abdominis	Peritoneum (diaphragm)	Abdominal wall	6 open myomeres
Rectus cervicis	Peritoneum (diaphragm)	Processus branchialis III and ceratobranchial IV	Two slips

### Larval cranium (Fig. 8)

**Fig. 8:**
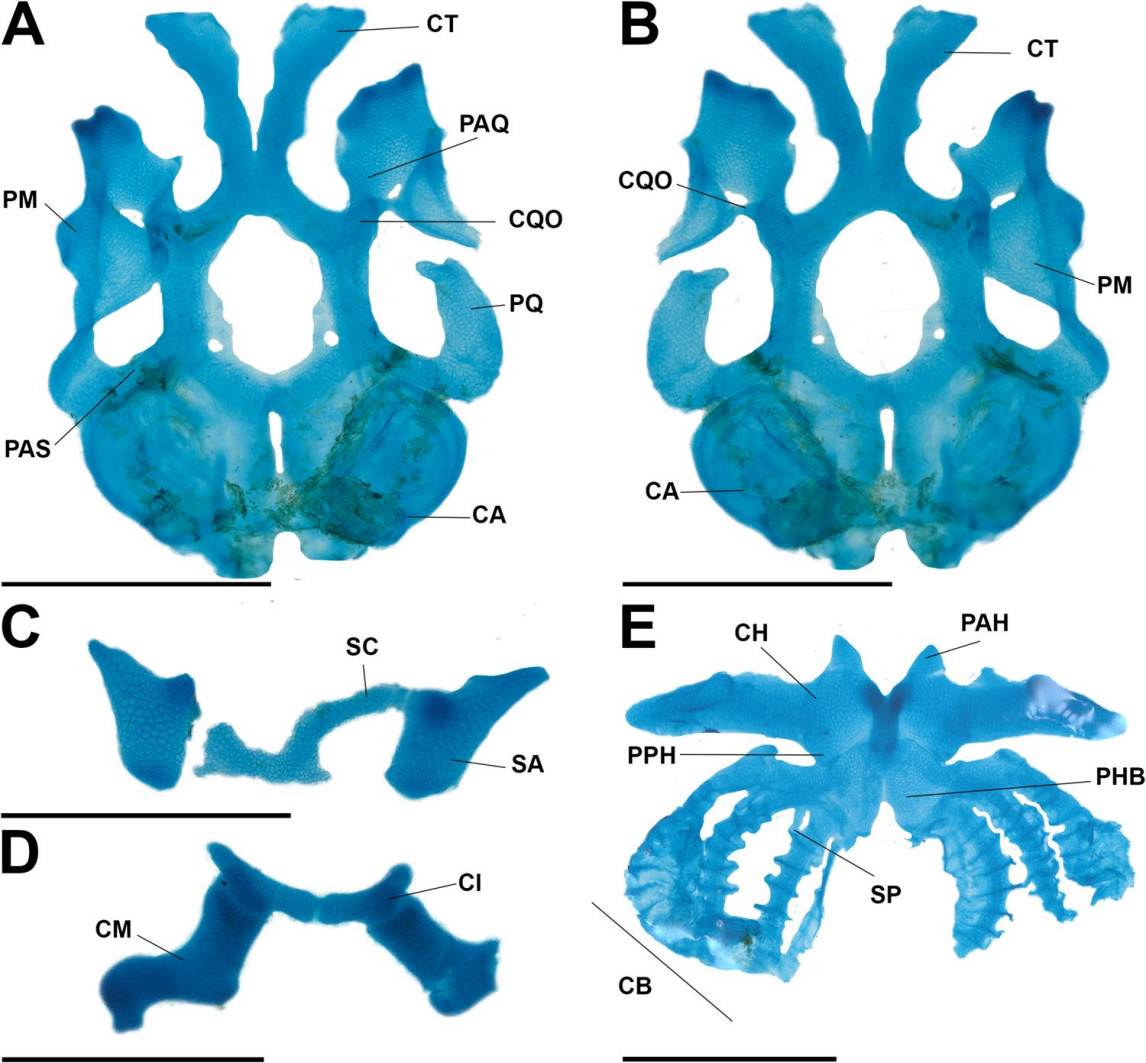
Cranial morphology of the tadpole of *Allophryne ruthveni* (ZMH 19226) at stage 30 in dorsal (**A**) and ventral (**B**) views; details of the suprarostral (**C**), infrarostralis and carilago Meckeli (**D**), and hyobranchial apparatus (**E**). CA, capsula auditiva; CB, ceratobranchials; CH, ceratohyal; CI, cartilago infrarostralis; CM, cartilago Meckeli; CQO, commissura quadratoorbitalis; CT, cornua trabeculae; PAH, processus anterior hyalis; PAQ, pars articularis quadrati; PAS, processus ascendens; PHB, planum hypobranchiale; PPH, procesus posterior hyalis; SA, suprarostral ala; SC, suprarostral corpus; SP, spiculae. Scale bar = 1.0 mm

Larval chondrocranium longer than wide (Fig. 8A, B); greatest width at the plane of the arcus subocularis (plane of processus ascendens). Suprarostral cartilage (Fig. 8C) composed of pars corporis and pars alaris; central corpora fused distally; corpus and ala fused proximally. Suprarostral corpus rectangular, with small projections on inner margin; suprarostral alae subtriangular, with well-developed processus anterior and posterior dorsalis*.* Cornua trabeculae short, V-shaped, medially divergent, uniform in most of its width, slightly wider distally; processus lateralis trabeculae present, small, triangular. Olfactory foramen elliptical. Fenestra basicranialis wide, very thin membrane present; caroticum et craniopalatinum foramina present, elliptical.

Cartilago orbitalis short and low; dorsal margin of foramen prooticus open. Opticum, oculomotorium, and prooticum foramina present; fisurra prootica elliptical; foramen opticum oval; foramen oculomorium rounded. Frontoparietal fontanelle large, almost elliptical; bordered laterally by taeniae tecti marginales, anteriorly by planum ethmoidale, and posteriorly by tectum synoticum; taenia tecti medialis and transversalis absent. Capsula auditiva rhomboid lacking the larval crista parotica. Jugulare and perilymphatic (superior and inferior) foramina present.

Palatoquadrate C-shaped; processus articularis short. Commissura quadratocranialis anterior thin, with a small, rounded processus quadratoethmoidalis; processus pseudopterygoideus absent. Processus muscularis triangular, tall, and thin; processus hyoquadrate evident ventrally. Processus antorbitalis short; commissura quadratoorbitalis present. Processus ascendens, rod-like, thin, and attached below the level of foramen oculomotorium (low attachment; see Sokol 1981; Haas 2003). Cartilago Meckeli (Fig. 8D) sigmoid, oriented perpendicular to the main axis of the chondrocranium, located ventral to cornua trabeculae. Cartilago infrarostralis rectangular, in V, joined medially by connective tissue.

Ceratohyals (Fig. 8E) long, flat, thin, subtriangular. Anterior margin bearing processus anterior hyalis and anterolateralis; processus anterior hyalis stout, with rounded borders. Condylus articularis well-developed, visible in ventral and dorsal views. Processus lateralis present, short, triangular. Posterior process present, triangular, large. Basihyal absent. Processus urobranchialis small, conical. Planum hypobranchiale, long, triangular, thin. Branchial basket has four curved ceratobranchials with numerous lateral projections, fused distally by commissura terminalis. Ceratobranchial I continuous with the planum hypobranchiale, bearing a tall and medially curved processus anterior branchialis. Ceratobranchials II and III are free from the planum and IV fused is in contact with planum hypobranchiale. Processus branchialis present at CB II and III, not contacting each other (open condition). Four spicules, curved.

## Discussion

### Reproductive biology

Frogs of the Allophrynidae lack basic information about their reproductive mode, developmental mode, and larval morphology (Altig 2018). Most published reports about their reproductive biology have been largely limited to anecdotal and sometimes conflicting observations (Caldwell 1996; Duellman 1997; Lescure and Marty 2000). In captivity, we found that *Allophryne ruthveni* exhibits an aquatic reproductive mode with indirect development.

The Allophrynidae has been consistently recovered as the sister family to glassfrogs of the Centrolenidae (e.g., Frost et al. 2006; Guayasamin et al. 2008; Pyron and Wiens 2011; Twomey et al. 2014; Streicher et al. 2018). However, the reproductive mode of these two families is notably different. Glassfrogs exhibit a semi-terrestrial reproductive mode, with eggs oviposited on arboreal vegetation and rocks over forested streams—embryos develop outside of water until hatching and then continue larval development underwater in streams (McDiarmid 1978; Wells 2007). Glassfrogs also lay eggs in a “clump” (sensu Altig and McDiarmid 2007) with extra-capsular jelly worked into the clutch, which can function to protect terrestrial embryos from dehydration and predation during development (Delia et al. 2020). In contrast, *A. ruthveni* exhibits aquatic oviposition, where single eggs are laid ungrouped within a simple jelly capsule—without any discernable extra-capsular jelly—and are scattered free in the water column before sinking to develop on benthic substrates. Furthermore, glassfrogs provide parental care to their eggs, which improves embryo survival in arboreal/terrestrial environments (McDiarmid 1978; Delia et al. 2017, 2020). In contrast, *A. ruthveni* parents did not interact with their eggs following oviposition nor did they associate with the same habitat as embryos in captivity.

Development is faster in *A. ruthveni* compared to glassfrogs from similar elevations/climates and/or under similar captive conditions. Under our laboratory conditions (22–24 °C), *A. ruthveni* embryos hatched 30–36 h after oviposition at Gosner (1960) stages 18–19 at the onset of a muscular response and a heartbeat. In contrast, embryonic development in glassfrogs—under similar conditions and/or from similar elevations/climates—lasts 7–21 days, with hatching occurring at stage 25 at the onset of feeding competence (Delia et al. 2019). In captivity, larval *A. ruthveni* required just 3–6 weeks to reach metamorphosis (with ample feeding), compared to 12–20 weeks for glassfrog tadpoles reared under similar laboratory conditions (including species of *Cochranella, Hyalinobatrachium, Sachatamia*, and *Teratohyla*; Delia and Taboada *unpublished data*). Taken together, our observations indicate that *A. ruthveni* exhibits a typical explosive-breeding life-history strategy, characterized by an aquatic reproductive mode and (relatively) rapid offspring development.

## Tadpole morphology

The Allophrynidae and the Centrolenidae have been consistently recovered as sister taxa, and this clade is frequently sister to Leptodactylidae (e.g., Frost et al. 2006; Pyron and Wiens 2011; Jetz and Pyron 2018). The lack of data for *Allophryne*, however, prevented the optimization of several character-states at that point of the anuran tree of life (e.g., Rada et al. 2019; Dias et al. 2020). Our data on the larval morphology of *Allophryne ruthveni* helps to shed light into the evolution of tadpoles in the Allophrynidae + Centrolenidae clade.

**Fig. 9:**
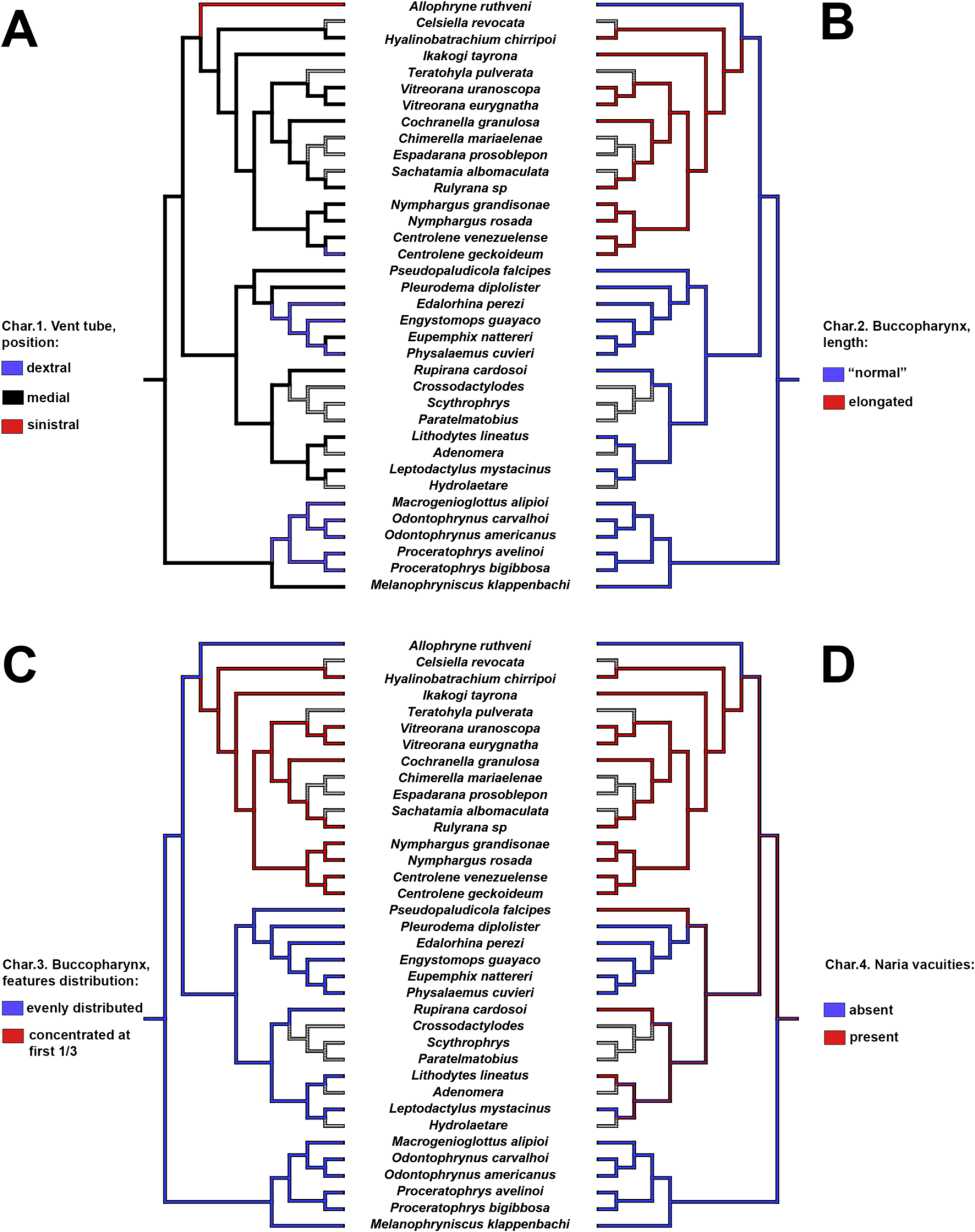
Parsimonious optimization of characters 1 (**A**), 2 (**B**), 3 (**C**), and 4 (**D**). Grey represents unknown/inapplicable condition

**Fig. 10:**
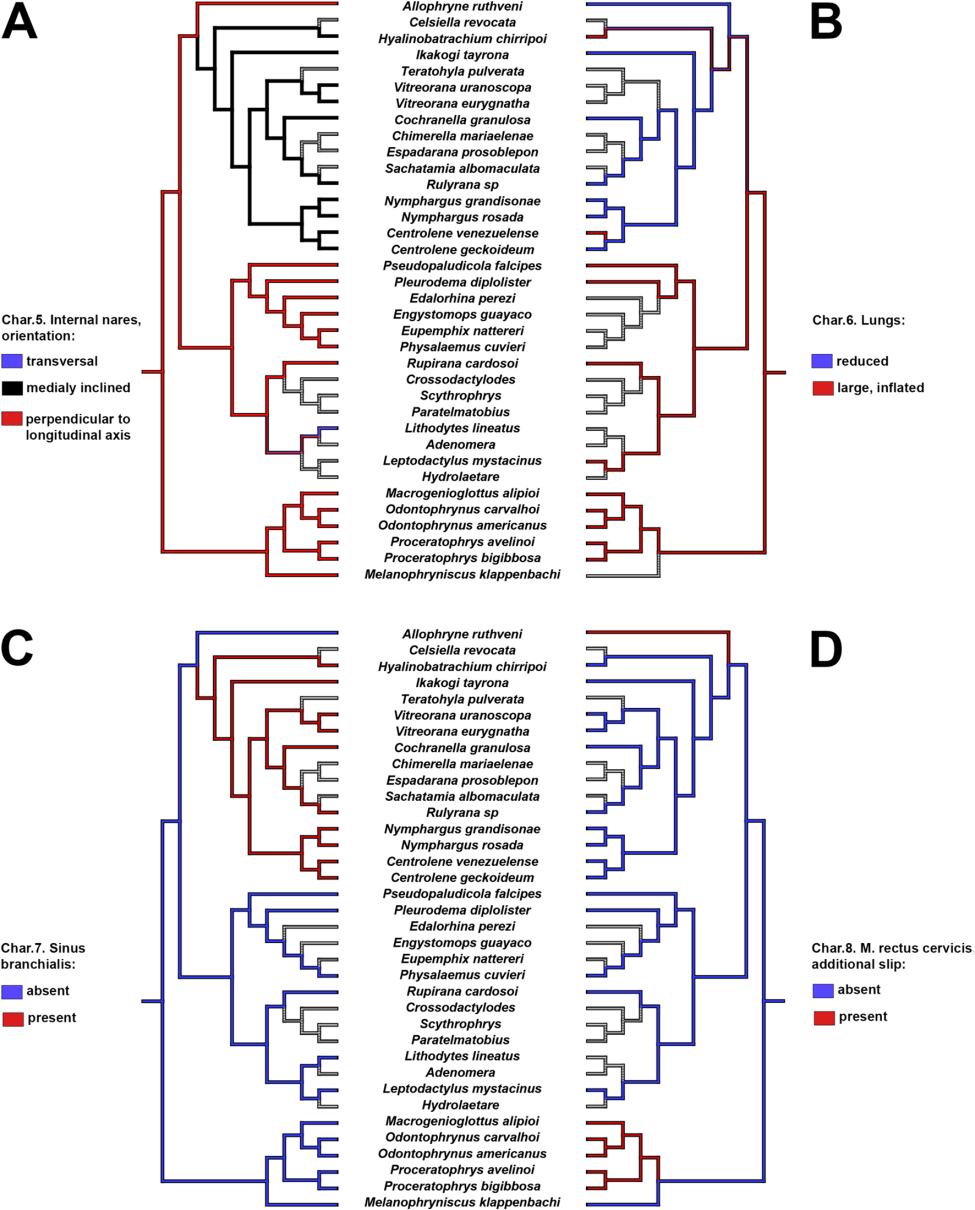
Parsimonious optimization of characters 5 (**A**), 6 (**B**), 7 (**C**), and 8 (**D**). Grey represents unknown/inapplicable condition

**Fig. 11:**
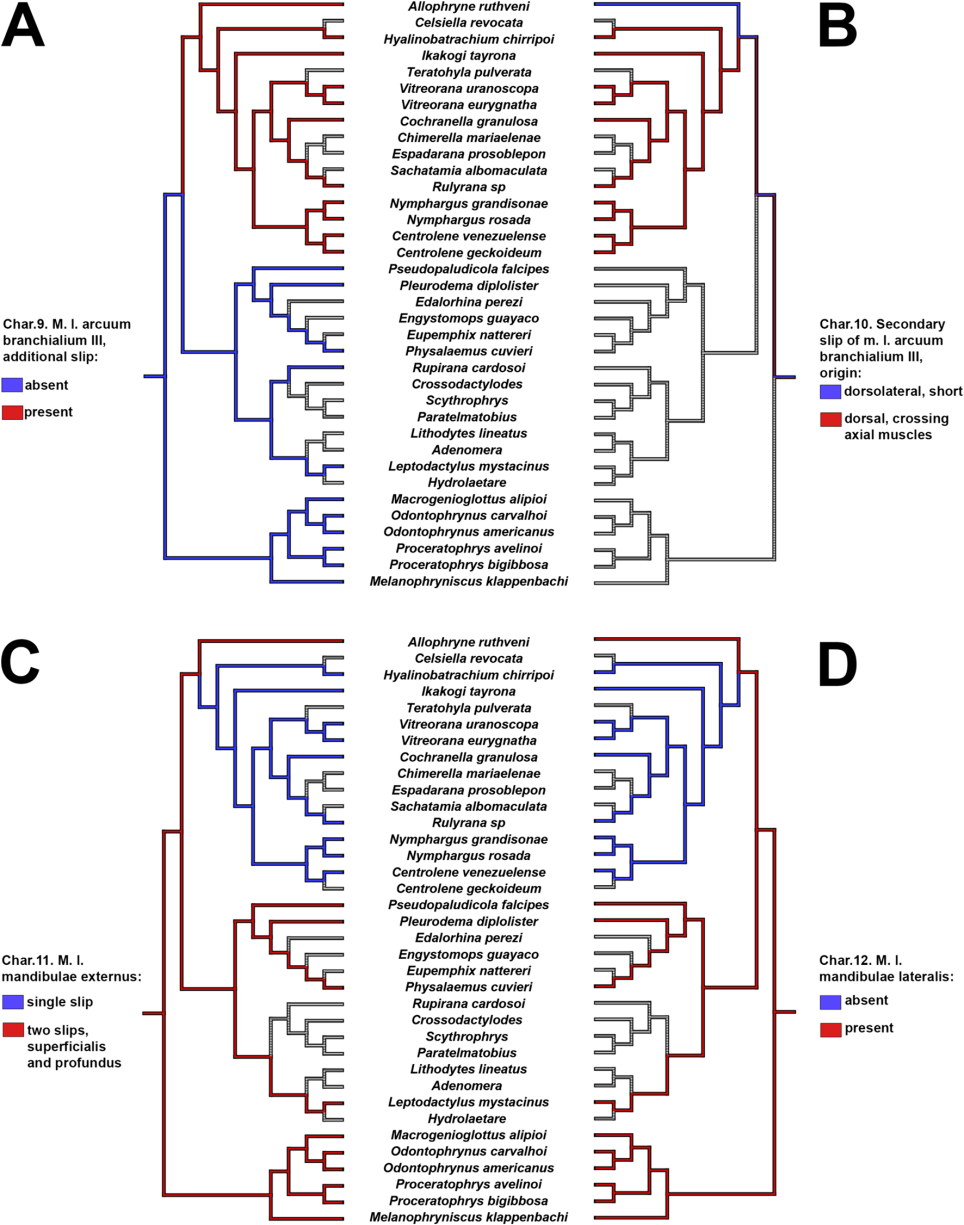
Parsimonious optimization of characters 9 (**A**), 10 (**B**), 11 (**C**), and 12 (**D**). Grey represents unknown/inapplicable condition

**Fig. 12:**
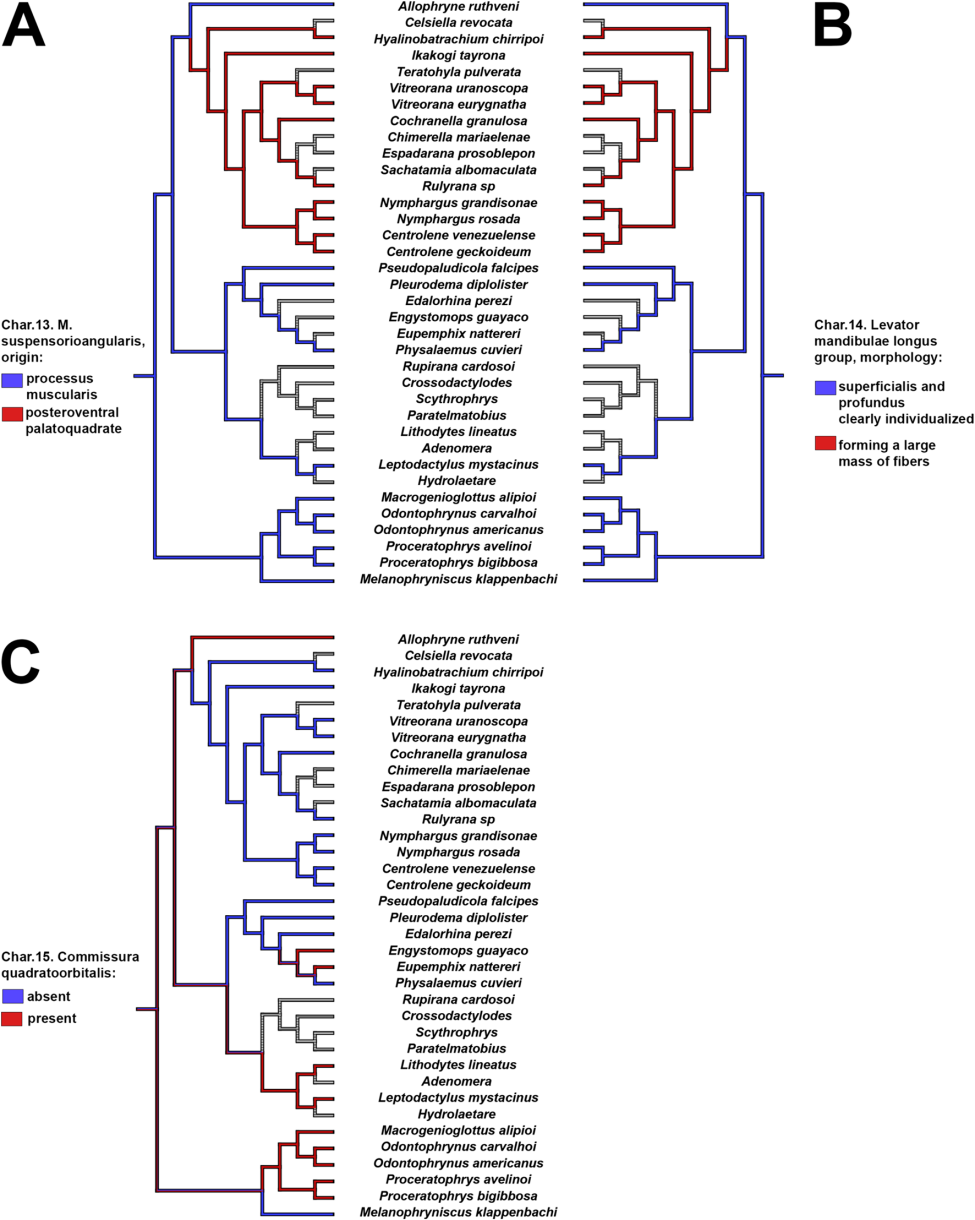
Parsimonious optimization of characters 13 (**A**), 14 (**B**), and 15 (**C**). Grey represents unknown/inapplicable condition

**Fig. 13:**
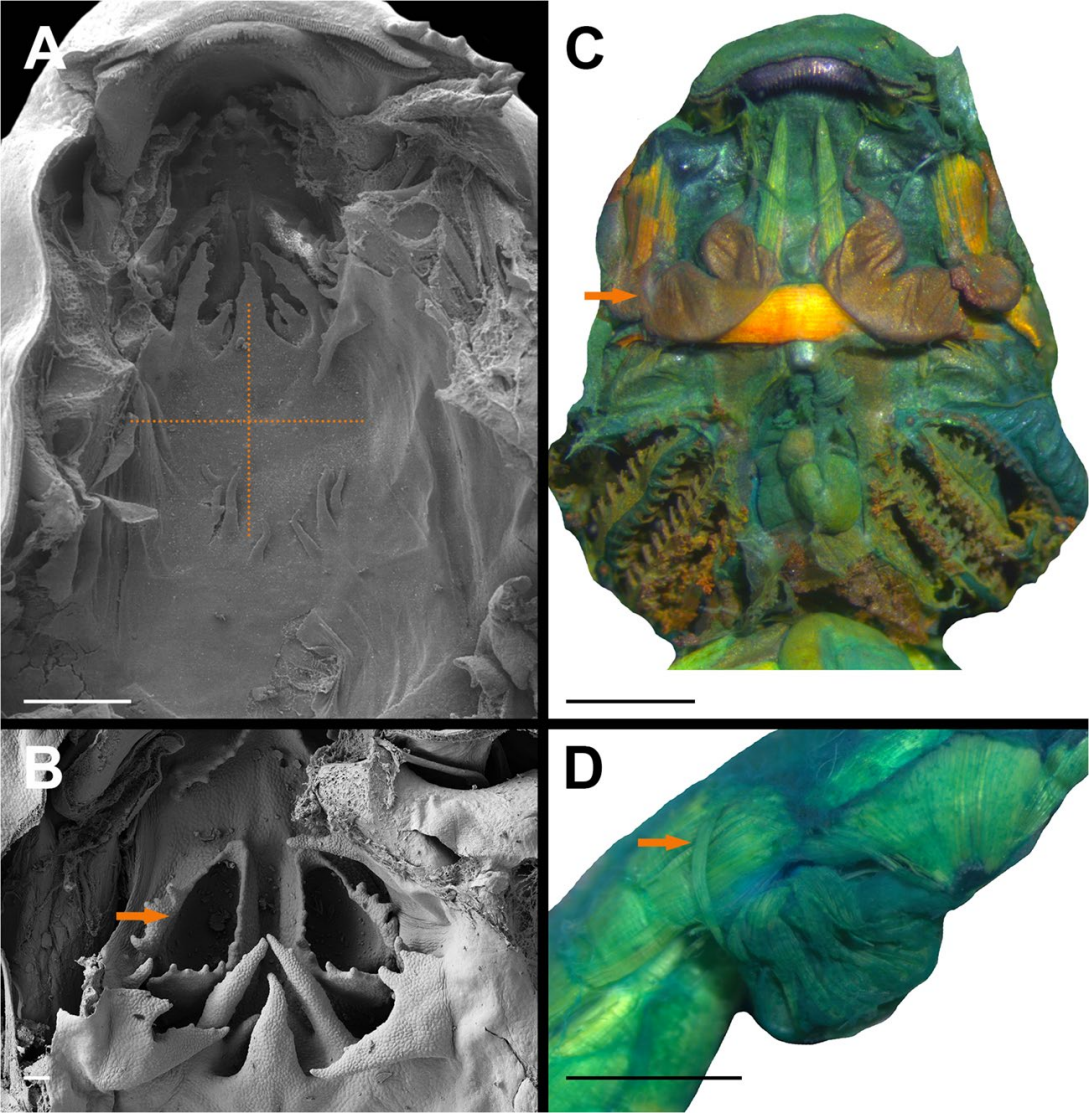
Phenotypic characters in centrolenid tadpoles. Buccal roof of *Ikakogi ispacue* (**A**), detail of the internal nares of *Centrolene venezuelensis* (**B**), sinus branchialis of *Rulyrana* sp. (**C**), and levator arcuum branchialium of *Vitreorana eurygnatha* (**D**). Scale bars = 500 µm (**A**), 100 µm (**B**), and 1.0 mm (**C** and **D**)Phenotypic characters in centrolenid tadpoles. Buccal roof of *Ikakogi ispacue* (**A**), detail of the internal nares of *Centrolene venezuelensis* (**B**), sinus branchialis of *Rulyrana* sp. (**C**), and levator arcuum branchialium of *Vitreorana eurygnatha* (**D**). Scale bars = 500 µm (**A**), 100 µm (**B**), and 1.0 mm (**C** and **D**)

Tadpoles of *Allophryne ruthveni* possess several plesiomorphic character-states shared with the close relatives of Allophrynidae + Centrolenidae (e.g., Leptodactylidae, Odontophrynidae + Bufonidae; Jetz and Pyron 2018). Nevertheless, several other characters are interesting in the evolution of *Allophryne* and Centrolenidae (Figs. 9, 10, 11, and 12; also see Appendix 2 for the complete list of characters), as discussed below (see also Fig. 13).

### External morphology

*Allophryne ruthveni* tadpoles have several characters observed in different lineages of frogs, such as an oval body, anteroventral oral disc, labial tooth row formula of 2(2)/3, body and tail skin pigmented—also observed in many leptodactylids, odontophrynids, and bufonids. The most remarkable feature of *A. ruthveni* tadpoles, however, is the presence of a sinistral vent tube. In most tadpoles, the vent tube is dextral or, sometimes, medial (Altig and McDiarmid 1999), and sinistral vent tubes have been reported a few times in the literature. For instance, a sinistral ventral tube was described for *Pleurodema thaul* (Cei 1980), *Lysapsus limellum* (Kehr and Basso 1990), *Gastrotheca manticola* (Duellman and Hillis 1987), and in some *Elachistocleis* species (Rossa-Feres and Nomura 2006). This character state, added to the labial tooth row formula, is diagnostic for *A. ruthveni*, an autapomorphy for the species, and a putative synapomorphy for the genus—the first larval phenotypic one.

### Buccopharyngeal cavity

Dias et al. (2020) enumerated 9 character states present in the buccopharyngeal cavity of centrolenids: (1) elongate buccal cavity, (2) with most structures concentrated anteriorly, (3) two pairs of infralabial papillae, (4) four lingual papillae, (5) presence of narial vacuities, (6) nares oriented longitudinally, (7) conical median ridge, (8) posterior-most pair of postnarial papilla aligned in row with median ridge, and (9) few pustulations (< 7) and papillae (< 10) on both floor and roof (Fig. 9A, B). They discussed, however, that some of these, such as the two pairs of infralabial and the four lingual papillae, were plesiomorphic and present in a more inclusive clade (Dias et al. 2020:120).

Our data for *Allophryne ruthveni* supports that these characters were plesiomorphic. *A. ruthveni* also possess two pairs of infralabial and the four lingual papillae. A large-scale analysis of the number of infralabial and lingual papillae is required to determine in which clade they optimize as synapomorphies.

The internal nares of *Allophryne ruthveni* are almost longitudinal; they are more angular than those of leptodactylids (e.g., Wassersug and Heyer 1988; Nascimento et al. 2021a, b) but not completely longitudinal as those of centrolenids (Wassersug 1980; Rada et al. 2019; Dias et al. 2020). Thus, the longitudinal orientation of internal nares is a putative synapomorphy of centrolenids.

The narial vacuities are a ciliated epithelium circumscribed by the internal nares and present in all centrolenids (Wassersug 1980; Rada et al. 2019; Dias et al. 2020; Fig. 9B), and in several leptodactylids (Nascimento et al. 2021a)—which might challenge the hypothesis of a synapomorphic condition in centrolenids (see Dias and Pie 2021). Well-defined vacuities are not observed in *Allophryne ruthveni*; however, they present an interesting morphology. The internal nares of this species are relatively enlarged, with the medial border bearing a densely ciliated epithelium. We interpret this condition as a not fully developed vacuity.

The ciliated epithelia concentrated in the medial portion of the internal nares are common in other anurans (Pedro H.S. Dias pers. obs.), but the narial vacuities are less frequent (Dias and Anganoy-Criollo 2024). We still consider the presence of vacuities as a putative synapomorphy of centrolenids, although we strongly suggest further studies regarding the proper definition of transformation series, state distribution, and optimization to support this hypothesis.

As in centrolenids, *Allophryne ruthveni* tadpoles have few pustulations and papillae in both roof and floor. We observed up to three buccal roof arena papillae and up to four buccal floor arena papillae in *A. ruthveni*, which is even less than that reported for some centrolenids (e.g., Dias et al. 2020). These papillae are well distributed in the buccopharyngeal cavity and the buccal roof and floor present a proportional distribution of features, contrasting with the elongated cavity of centrolenids, where most papillae are concentrated anteriorly. Whereas the elongation of the buccopharyngeal cavity seems to be a synapomorphy of the Centrolenidae, the reduction of papillation and other elements requires further studies in closely related lineages. Some leptodactylids, such as *Edalorhina perezi* (Nascimento et al. 2021b) also have few papillae in the buccal roof. An exploration of this character with a denser taxonomic sampling especially within leptodactylids is required to understand the evolution of the number and distribution of papillae in the buccopharyngeal cavity.

### Visceral components

Lungs were reduced in the studied larvae of *Allophryne ruthveni* (functionally absent, according to Haas 2003). Rada et al. (2019) reported the absence of lungs in tadpoles of *Ikakogi ispacue* and *I. tayrona*. This could suggest that the reduction of lungs represents a synapomorphy for Allophrynidae + Centrolenidae. Nevertheless, we observed large lungs in other centrolenids (e.g., *Espadarana prosoblepon*). This could represent a secondary gain of lung function within centrolenids, but the available data are insufficient to test this hypothesis. In addition, ontogenetic variation is not to be disregarded. Currently, there are no studies on the ontogeny of lungs in centrolenids because large developmental series are still scarce on collections; we advocate for further studies on lung morphology and development in that clade.

Another interesting visceral component is the sinus branchialis (Fig. 9C). This structure is formed by the circulatory system and lies ventrally to the hyobranchial apparatus (Hoffmann 2004, 2010). Hoffmann (2004) was the first to mention it in a tadpole description, and later he described it for several centrolenid tadpoles (Hoffmann 2010). Rada et al. (2019) suggested that the expanded sinus branchialis could have an involvement in respiratory performance in centrolenids. We did not observe the sinus branchialis in tadpoles of *Allophryne ruthveni*, but to date, it seems to be invariably present in centrolenids, representing a synapomorphy for the family.

### Larval muscles

The overall pattern of origin and insertion of the larval muscles in *Allophryne ruthveni* is similar to many lineages of anurans. Some traits, however, seem to be autapomorphic for the species, whereas others are shared with centrolenids.

*Allophryne ruthveni* tadpoles have an additional slip of the m. rectus cervicis inserting on the ceratobranchial IV. This condition was not observed in any centrolenid (Rada et al. 2019; this study), but it was described in the literature for many groups. Haas (2003) reported the presence of this additional slip in representatives of several lineages, such as *Litoria*, *Pyxicephalus*, *Tomopterna*, *Ptychadena*, *Limnonectes*, and *Scaphiophryne*. This slip is also present in most dendrobatoids (Haas 1995, 2003; Dias et al. 2021a). Conversely, it has been reported in several leptodactylids—*Physalaemus fernandezae*, *P. biligonigerus*, *Pleurodema kriegi* (Vera Candioti 2007; Barrasso et al. 2012) and in most *Leptodactylus* (Alcalde 2005)—but explicitly reported as absent in many others such *Physalaemus fernandezae* (Alcalde et al. 2006), *Pleurodema thaul,* and *Pleurodema bufoninum* (Barrasso et al. 2012), and in some *Leptodactylus* (Alcalde 2005; Vera Candioti et al. 2007; Grosso 2015). Such variation regarding the presence/absence of the additional slip of the rectus cervicis in leptodactylids makes it difficult to optimize these characters; it could be an autapomorphy of *A. ruthveni*, with independent gains in some leptodactylid lineages, or it could represent a synapomorphy for Leptodactylidae + (Allophrynidae + Centrolenidae), with a reversion in centrolenids. A more comprehensive dataset with broader taxonomic sampling within Leptodactylidae is required to test this hypothesis.

Rada et al. (2019) reported for *Ikakogi*, among other characters, the presence of a secondary slip of the m. levator arcuum branchialium III, that originated dorsally in the otic capsule, crossing the axial musculature, which we confirmed in all other examined centrolenids (i.e., some species of *Centrolene*, *Nymphargus*, *Rulyrana*, *Hyalinobatrachium* genera: MAR and PHD pers. obs.). We observed the same additional slip of that muscle in tadpoles of *Allophryne ruthveni*. However, it does not originate dorsally, nor does it cross the axial muscles. Instead, it originates dorsolaterally and is not related to the axial muscles. We recognize two transformation series (Hennig 1966; Grant and Kluge 2004); the additional slip of the levator arcuum branchialium III originated in the ancestor of Allophrynidae + Centrolenidae, and it is a synapomorphy for that clade, and in centrolenids, the muscle origin moved more dorsally, a synapomorphy for Centrolenidae. Rada et al. (2019) reported that the axial muscle is well developed in *Ikakogi*, reaching the anterodorsal portion of the otic capsule (Fig. 9D). This character state was also present in all other examined centrolenids (which optimizes as a synapomorphy for the family), contrasting with the condition observed in *A. ruthveni* tadpoles, in which the axial muscle insertion is restricted to the posteroventral region of the otic capsule (Fig. 7B).

Haas (2003) described the m. levator mandibulae externus in a single slip and subarcualis rectus II-IV inserting in the ceratobranchial III in the tadpoles of *Cochranella granulosa*. Regarding the number and insertion of the m. levator mandibulae externus, Rada et al. (2019) also observed the levator mandibulae externus in a single slip, as well as observed in other centrolenids. In tadpoles of *Allophryne ruthveni*, the m. levator mandibulae externus is present in two slips, with the superficialis inserting in the adrostral tissue mass, and the profundus inserting on the suprarostral ala. Given the presence of these two slips in leptodactylids (e.g., Haas 2003; Vera Candioti 2007; Vera Candioti et al. 2007), odontophrynids (e.g., Haas 2003; Gonzalez et al. 2014; Dias et al. 2019; Dias 2020), and bufonids (e.g., Haas 2003; Vera Candioti 2007; Aguayo et al. 2009; Haad et al. 2014), the loss of the secondary slip of the m. levator mandibulae externus is a putative synapomorphy of Centrolenidae.

Similar phenomena occurred with the muscle levator mandibulae lateralis. Haas (2003) reported this muscle as missing data for *Cochranella granulosa*, and Rada et al. (2019) cited explicitly as absent in *Ikakogi* tadpoles. This muscle is present in *Allophryne ruthveni* but invariably absent in centrolenids (PHD pers. obs. in several species of glassfrogs)—another putative synapomorphy for the family.

Regarding the insertion of the subarcualis rectus II-IV, *Allophryne ruthveni* retained the plesiomorphic condition (see Haas 2003), in which this muscle originates in the ceratobranchial IV and inserts on the ceratobranchial II. Rada et al. (2019) reported the same condition in *Ikakogi* larvae but, as Haas (2003) did, we observed further variation (e.g., it inserts on the ceratobranchial I in some *Centrolene*), and this character must be studied in more detail within centrolenids.

In *Allophryne ruthveni*, as in most anurans (e.g., Haas 2003; Vera Candioti et al. 2021; Dias et al. 2021a, b), the m. suspensorioangularis originates in the posterior margin of the processus muscularis. In all examined centrolenids, however, this muscle originates far posteriorly, in the ventral surface of the palatoquadrate, which we propose as a synapomorphy for Centrolenidae. This condition is very rare in anurans, being reported mainly in *Ascaphus* (Haas 2003), Alytidae (Haas 2003; Lukas 2021), and *Atelopus* (Haas 2003) larvae. Haas (2003) reported *Cochranella granulosa* as having this originating in the posterior margin of the processus muscularis, but the specimen examined by us had it as other centrolenids, in the ventral palatoquadrate.

Finally, the longus group of jaw levators is well-defined and individualized in *Allophryne ruthveni* tadpoles, whereas in centrolenids, they form a bulge, large mass of muscles (although individualized in their insertions). The condition observed in centrolenids is also a putative synapomorphy for the family.

### Larval cranium

Larval cranium of *Allophryne ruthveni* is quite similar to that of many leptodactylids (e.g., Larson and de Sá 1998; Vera Candioti et al. 2007), bufonids (e.g., Vera Candioti 2007; Aguayo et al. 2009), and odontophrynids (e.g., Nascimento et al. 2013; Dias et al. 2019; Dias 2020), and it is very different from that of centrolenids. Whereas centrolenids have an elongated cranium, with thin processus ascendens, enlarged pars articularis quadrati, and lack commissura quadratoorbitalis, *A. ruthveni* has a compact cranium, with a robust processus ascendens, a not so developed processus articularis. This suggests that the unique conditions observed in centrolenids are likely synapomorphies for that family.

Some of the conditions observed in *Allophryne ruthveni* might have broad implications for some major clades. For instance, the presence of commissura quadratoorbitalis, this cartilaginous structure bridges the processus muscularis and the processus antorbitalis, and it is present in several clades of frogs. More specifically, it has been consistently reported in leptodactylids (e.g., Larson and de Sá 1998; Nascimento et al. 2021a, 2022), bufonids (e.g., Vera Candioti 2007; Haad et al. 2014), and odontophrynids (e.g., Dias et al. 2013, 2014; Dias 2020). Dias et al. (2019) suggested that the presence of that commissura could be a synapomorphy of Odontophrynidae but given its presence in several closely related lineages (including Allophrynidae), it could be a synapomorphy of a more inclusive clade, and the absence of it a synapomorphy for Centrolenidae.

## Conclusions and remarks

In 2018, when discussing allophrynid frogs, Ronn Altig (Altig 2018:2) suggested that “One of two cases is likely: *Allophryne* has either a typical, pond-dwelling, hylid-like tadpole that no one has recognized or a centrolenid-like tadpole that has never been caught because people do not dredge in the bottom debris.” His comment was based on what Savage (1981:1183) called Starrett’s rule, which states that unique, specialized tadpoles metamorphose into ordinary frogs. *Allophryne ruthveni* is surely no ordinary frog, with a very interesting breeding biology, and its tadpoles, although at first glance seem like an ordinary “pond-like” tadpole, they are not. The tadpoles of *Allophryne ruthveni* have unique character states, such as the dextral vent tube, and they also have intriguing characters such as a reduced (but present) additional slip of the m. lab III. Our paper solved a hundred-year-old mystery but also raised several intriguing questions about the evolution and diversification of the Allophrynidae + Centrolenidae clade.

## Electronic supplementary material

Below is the link to the electronic supplementary material.

Supplementary file1 (MP4 44143 KB)

## Appendix 1 Examined material

All the material used in the present study is housed at the following: Celio F.B. Haddad collection, housed at the Universidade Estadual Paulista Julio Mesquita (CFBH), Instituto de Ciencias Naturales, Universidad Nacional de Colombia (ICN), Marco A. Rada, field series (MAR), Museu de Zoologia, Universidade Estadual de Feira de Santana (MZFS), Museu de Zoologia, Universidade de São Paulo (MZUSP), Museo de Zoología de la Pontificia Universidad Católica del Ecuador (QCAZ), Museu de História Natural, Universidade Federal de Alagoas (MHNUFAL), Universidade Federal do Mato Grosso (UFMT), and Zoological Museum Hamburg (ZMH).

**Table:**

Species	External	Buccopharynx	Muscles	Skeleton
Allophrynidae				
* Allophryne ruthveni*	ZMH 19225	ZMH 19224	ZMH 19226	ZMH 19226
Bufonidae				
* Melanophryniscus klappenbachi*	Baldo et al. (2014)	Baldo et al. (2014)	Baldo et al. (2014)	Baldo et al. (2014)
Centrolenindae				
* Centrolene gekoideum*	ICN 31096	ICN 31096	ICN 31096	ICN 31096
* Centrolene venezuelensis*	MAR 1387	MAR 1387	MAR 1387	MAR 1387
* Hyalinobatrachium chirripoi*	QCAZ 47332	QCAZ 47332	QCAZ 47332	QCAZ 47332
* I**kakogi tayrona*	Rada et al. (2019)	Rada et al. (2019)	Rada et al. (2019)	Rada et al. (2019)
* Nymphargus grandisonae*				
* Nymphargus rosada*	MAR662	MAR662	MAR662	MAR662
* Rulyrana* sp				
* Vitreorana eurygnatha*	MZUSP 80034	Dias et al. (2020)	MZUSP 80034	MZUSP 80034
* Vitreorana uranoscopa*	MZUSP 59946	Dias et al. (2020)	MZUSP 59946	MZUSP 59946
Leptodactylidae				
* Edalorhina perezi*	Nascimento et al. (2021b)	Nascimento et al. (2021a)		Nascimento et al. (2021a)
* Engystomops guayaco*	Nascimento et al. (2022)	Nascimento et al. (2022)		Nascimento et al. (2022)
* Eupemphix nattereri*	Vizotto (1973)	Perotti and Céspedez (1999)	-	F.A.C. Nascimento per. comm
* Leptodactylus mystacinus*	MZFS 1023	MZFS 1023	MZFS 1023	MZFS 1023
*Lithodytes lineatus*	Nascimento et al. (2021a)	Nascimento et al. (2021b)	Nascimento et al. (2021b)	-
* Physalaemus cuvieri*	UFMT 110	UFMT 110	UFMT 110	UFMT 110
* Pleurodema diplolister*	MZUSP 58997	MZUSP 58997	MZUSP 58997	MZUSP 58997
* Pseudopaludicola falcipes*	CFBH 213137	CFBH 213137	CFBH 213137	Alcalde and Barrasso 2013
Odontophrynidae				
* Macrogenioglottus alipioi*	MHNUFAL 10811	MHNUFAL 10811	MHNUFAL 10811	MHNUFAL 10811
* Odontophrynus americanus*	MZUSP 9570	MZUSP 9570	MZUSP 9570	MZUSP 9570
* Odontophrynus carvalhoi*				
* Proceratophrys avelinoi*	Dias et al. (2019)	Dias et al. (2019)	Dias et al. (2019)	Dias et al. (2019)
* Proceratophrys bigibbosa*	Dias (2018)	Dias (2018)	Dias et al. (2019)	Dias et al. (2019)

## Appendix 2 Character evolution

- Character 1. Vent tube, position: dextral (0), sinistral (1), medial (2).
- Character 2. Buccopharyngeal cavity, length: “normal” (0), elongate (1).
- Character 3. Buccopharyngeal cavity, elements: evenly distributed (0); concentrated on the anterior 1/3 (1).
- Character 4. Narial vacuities: absent (0); present (1).
- Character 5. Internal nares, orientation: transversal (0), medially inclined (1), perpendicular to the longitudinal axis (2).
- Character 6. Lungs: reduced (0); large, inflated (1).
- Character 7. Sinus branchialis: absent (0); present (1).
- Character 8. Musculus rectus cervicis, additional slip: absent (0), present (1).
- Character 9. Musculus levator arcuum branchialium III, secondary slip: absent (0), present (1).
- Character 10. Secondary slip of the musculus levator arcuum branchialium III, origin: dorsolateral, short (0); dorsal, crossing the axial musculature (1).
- Character 11. Musculus levator mandibulae externus, slips: single slip (0), divided in two slips, superficialis and profundus (1).
- Character 12. Levator mandibulae lateralis: absent (0), present (1).
- Character 13. Musculus suspensorioangularis, origin: at the posterior margin of processus muscularis (0), posteroventral palatoquadrate (1).
- Character 14. Levator mandibulae longus group, morphology: superficialis and profundus clearly individualized (0), forming a large mass of fibers (1).
- Character 15. Commissura quadratoorbitalis: absent (0), present (1).
